# Statistical Parametric Mapping and Voxel-Based Specific Regional Analysis System for Alzheimer’s Disease (VSRAD): Principles and Clinical Applications

**DOI:** 10.3390/brainsci15090999

**Published:** 2025-09-16

**Authors:** Shinji Yamamoto, Nobukiyo Yoshida, Noriko Sakurai, Yukinori Okada, Norikazu Ohno, Masayuki Satoh, Koji Takeshita, Masanori Ishida, Kazuhiro Saito

**Affiliations:** 1 Department of Radiological Technology, JCHO Tokyo Yamate Medical Center, Shinjuku 169-0073, Japan; 2 Department of Radiological Technology, Faculty of Medical Technology, Niigata University of Health and Welfare, Niigata 950-3198, Japan; 3 Department of Radiological Science, Faculty of Medical Science and Technology, Gunma Paz University, Takasaki 370-0006, Japan; 4 Department of Radiology, Tokyo Medical University, Tokyo 160-0023, Japan; 5 Graduate School of Medical Science, Suzuka University of Medical Science, Suzuka 513-8670, Japan; 6 Department of General Medicine, Iga City Genera Hospital, Iga 518-0823, Japan; 7 Ohno Medical Clinic, Iga 518-0216, Japan; 8 Center for Comprehensive Care and Research on Memory Disorders, National Center for Geriatrics and Gerontology, Obu 474-8511, Japan; 9 Department of Radiology, JCHO Tokyo Yamate Medical Center, Shinjuku 169-0073, Japan

**Keywords:** dementia, Alzheimer’s disease, voxel-based morphometry

## Abstract

**Background**: The voxel-based specific regional analysis system for Alzheimer’s disease (VSRAD) allows quantitative evaluation of the degree of an individual’s brain atrophy through statistical comparison of brain magnetic resonance imaging (MRI) of their brain to a normative database of healthy Japanese individuals. Currently, the VSRAD is used in routine clinical practice in the diagnosis of Alzheimer’s disease (AD) and dementia with Lewy bodies (DLB). Recent studies using VSRAD have explored its utility in the assessment of brain atrophy associated with various conditions, including diabetes, oral health status, and olfactory dysfunction. This review summarizes the principles of the VSRAD and its foundational method, voxel-based morphometry (VBM), and their clinical and research applications. **Methods**: This narrative review was conducted by performing a literature search of PubMed to identify articles regarding VBM and the VSRAD that were published between 2005 and 2025. **Results**: VSRAD yields four indices for quantifying the severity and extent of gray matter atrophy, especially in the medial temporal lobe. Studies have demonstrated its high diagnostic accuracy in distinguishing among AD, mild cognitive impairment (MCI), and DLB. Furthermore, it is correlated with neuropsychological test scores and has been applied to evaluate brain changes associated with diabetes, olfactory dysfunction, and physical inactivity. Motion-corrected MR images, which utilize AI techniques, have also been validated using VSRAD-derived metrics. **Conclusions**: Quantifying brain atrophy using the VSRAD allows objective evaluation and facilitates the investigation of its association with various diseases. Specifically, VSRAD can be considered a useful adjunctive tool for diagnosing AD and DLB.

## 1. Introduction

Dementia is characterized by loss of cognitive function. Its diagnosis usually involves neuropsychological assessments, including the revised Hasegawa Dementia Scale (HDS-R), Clinical Dementia Rating (CDR), and Mini-Mental State Examination (MMSE), with critical support from neuroimaging modalities. HDS-R is only used in Japan, where it was developed. Magnetic resonance imaging (MRI) offers high contrast resolution without exposure to radiation and can provide anatomical and functional information regarding the brain. In clinical practice, MRI is used in the diagnosis of mild cognitive impairment (MCI) and Alzheimer’s disease (AD). Specifically, MCI and AD are characterized by disease-specific hippocampal atrophy [1]. Accordingly, dementia can be diagnosed through visual assessment of brain morphology; however, this is subjective and dependent on the experience of radiologists and neurologists. Therefore, there is a need for objective and quantitative tools for assessing brain atrophy. The voxel-based specific regional analysis system for Alzheimer’s disease (VSRAD) is a software used to quantify brain atrophy by comparing MRI of a patient’s brain with a normative database of healthy Japanese individuals [2]. The VSRAD was developed under the leadership of Hiroshi Matsuda. Specifically, the VSRAD was co-developed by Dai Nippon Printing Co., Ltd. (Tokyo, Japan) and Eisai Co., Ltd. (Tokyo, Japan). It is currently being distributed by Eisai Co. Since February 2020, VSRAD has been officially approved as a medical device and is widely used in clinical practice throughout Japan. Specifically, it is widely used in clinical practice to support the diagnosis of AD and dementia with Lewy bodies (DLB). This review aims to summarize the principles of the VSRAD and its foundational method, voxel-based morphometry (VBM), and their clinical and research applications. In this review, we focused on the utility of the VSRAD primarily for AD, DLB, and MCI, while also reporting on its application to other diseases.

## 2. Materials and Methods

### 2.1. Literature Selection Process

In this narrative review, we performed a literature search of PubMed to identify articles related to VSRAD published between 2005 and 2025. The key search terms included “VSRAD, VBM, MRI”, and other related terms. We primarily included original research articles regarding VSRAD. We excluded case reports, ambiguous content, narrative reviews, meta-analyses, systematic reviews, articles written only in Japanese, publications from inaccessible journals, and articles that could not be directly accessed. However, we included an important review written by the developer of VSRAD, Dr. H. Matsuda [2]. Additionally, one article authored by YO, not indexed in PubMed, was included due to its relevance. We also selected key articles related to VBM where appropriate. To describe the technical principles of VSRAD and VBM, we referred to relevant textbooks published in Japan during the same period (2005–2025). Given that references related to VSRAD spanned various sources, we obtained citation permissions from KANEHARA Co., Ltd. (Tokyo, Japan), Medical View Co., Ltd. (Tokyo, Japan), and Nagai Shoten Co., Ltd. (Osaka, Japan). Further approval was granted by Eisai Co., Ltd. (Tokyo, Japan), the distributor of VSRAD.

**Figure 1:**
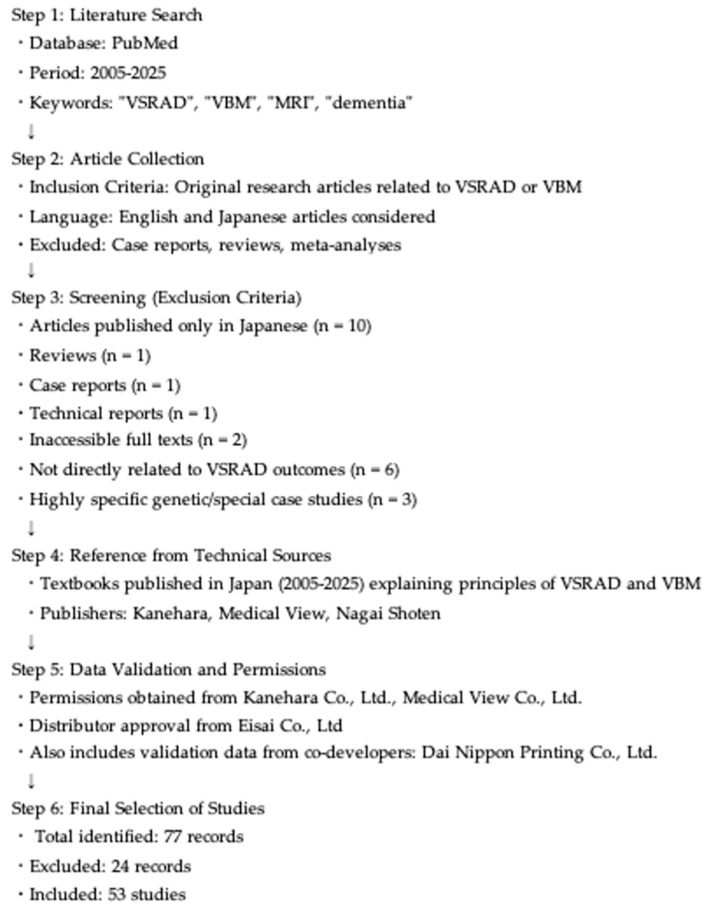
Flowchart of the literature selection process for the VSRAD review. The process included a PubMed search (2005–2025), application of inclusion and exclusion criteria, reference to technical textbooks published in Japan, and validation using data from the developer/distributor. Eligible original studies and references were synthesized narratively in this review.

Regarding music therapy, both VSRAD and VBM were employed in a study conducted by one of the authors (Sato). Therefore, we also included VBM-related findings in this review. Given the limited number of eligible studies and broad thematic scope, a systematic review or meta-analysis was not feasible. Instead, we adopted a narrative review methodology. Figure 1 illustrates the literature selection process and flowchart for the VSRAD review.

Overall, the following inclusion criteria were applied: (1) original clinical studies using VSRAD as a primary or adjunctive tool for the evaluation of AD, MCI, DLB, or related dementias, (2) articles published in peer-reviewed journals, and (3) full-text availability in English. The following exclusion criteria were applied: (1) case reports; (2) narrative reviews, systematic reviews, or meta-analyses; (3) technical reports or phantom-only studies without direct clinical outcomes; (4) publications available only in Japanese without an English abstract or full text; (5) articles for which the full text was inaccessible; and (6) studies not directly related to VSRAD or dementia.

After applying these criteria, a total of 53 original studies were included in this narrative review. The PRISMA-like flow diagram in Figure 1 illustrates the screening process, including the number of records identified, screened, excluded, and included, with exclusion categories explicitly noted (*n* = 24 excluded, *n* = 53 included).

### 2.2. Fundamentals of VBM and VSRAD

Recent advances in brain structural analysis have allowed quantitative evaluation of brain morphology and volume by defining regions of interest (ROIs). Based on the study by Aoki and Kasai [3], we describe the principles of VBM and statistical parametric mapping (SPM). VBM is widely used to statistically analyze the density and volume of gray and white matter on a voxel-wise basis. It automatically quantifies grey matter (GM), white matter, and cerebrospinal fluid volumes based on MRI T1-weighted images [4].

VBM is typically performed using SPM, which is a statistical image analysis software package developed at University College London that runs in MATLAB MathWorks (MATLAB RUNTIME Library, version R2013a, Natick, MA, USA), The latest version, SPM12, supports both structural and functional brain image analysis. Here, MRI data are first converted from the Digital Imaging and Communications in Medicine (DICOM) format to the Neuroimaging Informatics Technology Initiative format, which is the standard for brain imaging research [5]. In the preprocessing pipeline of VBM using SPM12, segmentation is performed to extract GM, white matter, and cerebrospinal fluid volumes from whole-brain images. Next, spatial normalization is performed to align the individual brains to a standardized anatomical space, followed by smoothing to reduce inter-individual variability and prepare data for statistical testing. Subsequently, voxel-wise statistical analyses, including global normalization and construction of design matrices based on the general linear model, are performed. Finally, results are visualized by superimposing them onto the brain surfaces or sectional images for easier interpretation.

VBM-based studies often employ longitudinal designs to assess temporal changes in GM volume; additionally, they typically utilize age-matched controls. For example, two MRI scans were performed over a 4-month interval in 40 healthy young adults to assess the effects of sex and brain-derived neurotrophic factor genotype on GM volume. Although the overall changes were minimal and stable over a short period, the volume variability was significantly influenced by sex and genotype, especially in the “Female–Val66Val” group [6,7]. Notably, SPM is highly suitable for statistical analyses involving large cohorts but not for individual case evaluations. Additionally, SPM assumes a normal distribution of voxel values in the reference dataset and therefore requires a dataset obtained from ≥30 healthy controls.

### 2.3. Applications of VSRAD

The principles underlying VSRAD are primarily based on the work of Matsuda et al. [8,9,10,11,12,13]. VSRAD utilizes either sagittal 3D T1-weighted images in DICOM format with a slice thickness of 1.0–1.5 mm or axial whole-brain 3D T1-weighted images specifically formatted for analysis. Initially, all images are inspected to ensure there are no artifacts or other factors interfering with the analysis. Subsequently, the images are loaded and resampled to isotropic voxel dimensions (e.g., 1.0 × 1.0 × 1.0 mm or 1.5 × 1.5 × 1.5 mm) across the X-, Y-, and Z-axes. Subsequently, a linear transformation is applied to standardize the head size and align the anterior commissure–posterior commissure line to a horizontal orientation. Non-brain regions are excluded. Furthermore, if the skull exhibits a higher signal intensity than the brain parenchyma due to its fat content, the skull signal is suppressed. MRI data are automatically segmented into GM, white matter, and cerebrospinal fluid. Subsequently, the GM and white-matter images are normalized to a standard brain template and smoothed using an 8 mm Gaussian kernel. This smoothing process reduces interindividual variability in brain functional localization, improves the signal/noise ratio, and allows approximation of the intensity distribution to a normal distribution. Subsequently, the brain morphology of the individual is deformed to match that of the standard brain template. Next, the absolute values of the GM density and volume for each voxel are statistically compared to a normative database of healthy controls. The standard database included in VSRAD comprises data from 80 healthy individuals aged 54–70 years. Furthermore, an additional database comprising 232 healthy individuals aged 50–89 years, developed by the University of Tsukuba, may also be incorporated.

### 2.4. Z-Score and Quantitative Analysis of VSRAD

In this comparison, VSRAD performs voxel-wise statistical analyses using Z-scores, which are calculated as follows (Formula (1)):

(1) $$Z-score=\frac{\left(mean\;voxel\;value\;\left[normal\right]\;-\;mean\;voxel\;value\;\left[patient\right]\right)}{standard\;deviation\;\left[normal\right]}$$

The Z-score is a statistical measure that indicates the deviation of an individual’s voxel value from the mean value of healthy controls, which is expressed in standard deviation units. For example, a Z-score of 2 indicates a deviation greater than two standard deviations from the mean, which corresponds to a statistically significant difference at *p* < 0.05. VSRAD operates on the assumption that the individual’s brain image has the same volumetric properties as that of a healthy individual. Voxels with a Z-score ≥ 2 are considered to indicate significant atrophy (*p* < 0.05) and are visually highlighted on the individual’s brain image. Specifically, the Z-score distribution obtained using the standard template is mapped back onto the individual brain image by inversely applying the deformation parameters applied during anatomical normalization. This facilitates visual evaluation of regions with significant atrophy (high Z-scores). Although conventional VBM analysis typically requires MATLAB, VSRAD has been developed to perform equivalent processing independently of MATLAB under licensing from University College London.

Quantitative analysis using VSRAD is primarily based on the work by Matsuda et al. [8,9,10,13]. Here, a volume of interest (VOI) is defined in the hippocampus and parahippocampal gyrus, which characteristically exhibit atrophy in AD, to evaluate localized brain volume. VSRAD provides four primary indices for analysis:

1. Severity of VOI atrophy: This index represents the mean Z-score within the VOI, with higher values indicating more severe atrophy. A Z-score of 0–1 indicates minimal or no atrophy; 1–2 indicates mild atrophy; 2–3 indicates moderate atrophy, and >3 indicates severe atrophy;
2. Extent of VOI atrophy: This refers to the percentage of the VOI with a Z-score ≥ 2. Proportions of 0–30%, 30–50%, and >50% are indicative of localized, moderate, and widespread atrophy, respectively;
3. Extent of GM atrophy: This index represents the proportion of the entire GM volume with a Z-score ≥ 2. A value exceeding 10% suggests significant global GM atrophy;
4. Ratio of VOI to GM atrophy: This ratio reflects the extent of atrophy within the VOI relative to the entire brain. Higher values indicate greater selectivity for atrophy within the VOI. Specifically, ratios of 0–5, 5–10, and >10 indicate no, moderate, and high selectivity, respectively.

### 2.5. Development of VSRAD

The history of VSRAD is described primarily based on the work of Matsuda [8]. VSRAD was first introduced in 2005. In 2009, an updated version called VSRAD Plus was released, which featured improvements to the display interface. This was followed by VSRAD Advance in 2012, which incorporated enhanced image-processing capabilities. In 2015, VSRAD Advance 2 was launched, which improved the differential diagnosis between AD and DLB.

VSRAD Advance adopts the diffeomorphic anatomical registration through exponentiated lie algebra (DARTEL) method, which enables more accurate anatomical normalization. DARTEL enables high-dimensional nonlinear transformations using a large number of parameters, and therefore facilitates more precise alignment of GM and white matter to a standard brain template. Earlier versions of SPM applied discrete cosine transform-based normalization, which is less effective in patients with widened cerebral sulci and often results in inadequate morphological alignment and misclassification of GM as an atrophied tissue. Accordingly, the introduction of DARTEL has significantly improved the accuracy of GM evaluation [8]. Furthermore, DARTEL allows improved reliability of anatomical normalization of white matter in individuals with enlarged ventricles [8]. Taken together, DARTEL has minimized the influence of interindividual anatomical variability, facilitating accurate structural assessment of both GM and white matter [10].

### 2.6. Use of 3T RI

The VSRAD database was originally constructed using images acquired with 1.5 Tesla (T) MRI systems. Although VSRAD is compatible with images obtained from 3T MRI scanners, there are variations in certain quantitative indices depending on the field strength. Specifically, when using 3T MRI, the average severity and extent of VOI atrophy are ≈10% and ≈15% lower than those in 1.5T MRI. Conversely, the extent of GM atrophy tends to be ≈30% higher while the ratio of VOI/GM atrophy is ≈30% lower in 3T MRI than in 1.5T MRI. Therefore, the threshold values should be adjusted to account for these differences when interpreting VSRAD results obtained from 3T MRI data [10,13,14].

### 2.7. Early-Onset Dementia

Among patients with early-onset and late-onset AD (onset at age ≤ 65 (*n* = 18) and ≥66 years (*n* = 80), respectively), the mean Z-scores calculated using the VSRAD were 1.83 ± 0.92 and 2.90 ± 1.40, respectively. This suggests that, when the analysis is limited to the medial temporal lobe, VSRAD may underestimate the degree of atrophy in early-onset cases [13,15].

### 2.8. VSRAD and Artifact Images

Yoshida et al. used the VSRAD to evaluate a motion-correction technique for MRI images based on machine learning [16]. They used motion-free MR images to generate synthetic motion artifacts by modifying k-space data. These artifact-containing images were paired with their original motion-free counterparts and used as inputs to train the machine learning model. For validation, additional images with synthetic motion artifacts were generated and processed using the model to obtain motion-corrected outputs. Evaluation of the corrected images was performed using three methods:

1. Visual assessment by two radiologists;
2. Quantitative image quality metrics, including the peak signal/noise ratio and structural similarity index measure;
3. Changes in Z-scores obtained through VSRAD analysis.

Analysis of uncorrected motion artifact images with VSRAD yielded elevated Z-scores, which potentially overestimated brain atrophy, especially in the hippocampus. Contrastingly, Z-scores from the machine learning-based images were closer to those of the original motion-free images, which suggests a more accurate assessment of hippocampal atrophy. Taken together, VSRAD can serve as a valuable tool for evaluating the efficacy of motion correction techniques for MRI based on machine learning [16].

### 2.9. VSRAD in Clinical Practice

The VSRAD received approval as a medical device in March 2020 and is currently used as a clinical support software in daily practice across medical institutions in Japan.

## 3. Diagnostic Accuracy of the VSRAD

### 3.1. Diagnostic Accuracy for Alzheimer’s Disease

A study conducted in 2005 evaluated the diagnostic performance of the VSRAD in a cohort comprising 30 patients with AD and 41 healthy controls, reporting a diagnostic accuracy of 87.8% in differentiating AD from normal aging [17]. A more recent study assessed the diagnostic accuracy of VSRAD Advance in 45, 30, and 41 patients with very mild, mild, and moderate-to-severe AD, respectively, as well as in 40 healthy controls. They found that the diagnostic accuracy of VSRAD Advance was 91.6%, 95.8%, and 98.2% for very mild, mild, and moderate-to-severe cases, respectively [18]. This demonstrates consistently high diagnostic performance across all disease stages.

Another study compared the visual rating scores (VRS) and VSRAD scores among 37 patients with AD (14 males, 23 females; age: 78.8 ± 8.8 years), 29 patients with MCI (9 males, 20 females; age: 76.0 ± 6.7 years), and 21 healthy controls (6 males, 16 females; age: 72.9 ± 6.7 years). The VRS score, which evaluates the degree of brain atrophy from 0 to 4, was superior to the VSRAD score in distinguishing AD from MCI [19]. Contrastingly, the VSRAD score is superior to the VRS score in distinguishing MCI from healthy controls [19].

### 3.2. Diagnostic Performance of VSRAD for Dementia with Lewy Bodies

#### 3.2.1. Atrophy in the Entorhinal Cortex Atrophy

In a study involving 60 patients, each with AD and DLB, the DLB group exhibited statistically significantly lower Z-scores compared to the AD group (2.25 ± 1.10 vs. 2.85 ± 1.33) [20].

#### 3.2.2. Atrophy in the Dorsal Brainstem

A study involving 30 patients with AD and 60 patients with DLB identified several brain regions that were useful for differentiating between AD and DLB. Specifically, patients with DLB demonstrated characteristic atrophy in the right dorsal midbrain, right dorsal cerebrum, and bilateral cerebellum [21]. When the midbrain was designated as the VOI, the area under the receiver operating characteristic curve (AUC) was 0.75, which indicates moderate diagnostic performance [21].

#### 3.2.3. Evaluation of Dorsal Brainstem Atrophy Using the VSRAD

A large-scale study involving 385 patients with AD and 239 patients with DLB proposed the following VSRAD-based diagnostic criteria [22]:

1. The case is classified as AD when the severity of VOI atrophy is ≥2.185;
2. The case is classified as DLB when the severity of VOI atrophy is <2.185, the ratio of GM atrophy in the dorsal brainstem to that in the medial temporal lobe is ≥0.195, and the ratio of white matter atrophy in the dorsal brainstem to that in the medial temporal lobe is ≥0.195;
3. All other patients were classified as having AD.

When applying criterion 2 alone, the sensitivity, specificity, and overall diagnostic accuracy for DLB were 56.3%, 68.3%, and 63.3%, respectively [22].

VSRAD Advance 2 facilitates the evaluation of the likelihood of AD based on ROIs in the hippocampus and surrounding medial temporal structures; moreover, it allows assessment of potential DLB by including the dorsal brainstem as an ROI. Specifically, a severity score ≥2.0 for VOI atrophy is suggestive of AD. Contrastingly, DLB should be considered if the severity score is <2.0 and both the GM and white matter dorsal brainstem/medial temporal lobe VOI atrophy ratios are ≥0.2.

Moreover, Okada et al. reported a negative correlation (r = −0.40) between whole-brain and dorsal brainstem GM atrophy in 25 patients with AD (6 males, 19 females; mean age: 81.6 ± 5.5 years). Contrastingly, there was a positive correlation (r = 0.78) between whole-brain GM atrophy and dorsal brainstem white matter atrophy in 11 patients with DLB (five males, six females; mean age: 81.3 ± 6.2 years) [23].

## 4. Comparison Between the VSRAD and Cerebral Blood Flow SPECT

Both the VSRAD and cerebral blood flow SPECT provide complementary perspectives in dementia imaging: the VSRAD highlights structural atrophy in the medial temporal lobe, whereas SPECT, particularly with eZIS analysis, reflects functional perfusion changes such posterior cingulate and parietotemporal hypoperfusion. Therefore, direct comparisons and combined analyses of these modalities are clinically meaningful, especially for differential diagnosis between AD and DLB.

### 4.1. Supporting Dementia Diagnosis Using eZIS and the VSRAD

In Japan, cerebral blood flow scintigraphy is reimbursed by insurance and commonly used as a diagnostic imaging modality for dementia. This method utilizes single-photon emission computed tomography (SPECT) to generate brain tomographic images, which allows evaluation of characteristic patterns of cerebral blood flow reduction to support differential diagnosis. Several studies have compared the VSRAD and cerebral blood flow SPECT. Specifically, when using SPECT with 99mTc-ECD, a specialized software known as the easy Z-score Imaging System (eZIS) allows statistical comparison of a patient’s cerebral perfusion map with a normative database using Z-score analysis. The eZIS was primarily developed by Dr. Hiroshi Matsuda and colleagues, and it is widely used to facilitate the diagnosis of AD and DLB in clinical practice.

#### 4.1.1. Comparison with Cerebral Blood Flow

A study involving 10 patients with MCI and 16 patients with AD investigated the relationship between the VSRAD and cerebral blood flow SPECT [24]. This study utilized 99mTc-ECD as the radiopharmaceutical agent. Correlation analyses between VSRAD-derived Z-scores in the entorhinal cortex and regional cerebral blood flow values revealed the following findings: r = −0.548 in the right thalamus, r = −0.593 in the left hippocampus, and r = −0.630 in the right hippocampus. There were significant negative correlations between the Z-scores and cerebral blood flow in the right cerebral hemisphere (r = −0.398), left thalamus (r = 0.497), left parietal lobe (r = −0.450), right parietal lobe (r = −0.503), and right cerebellum (r = 0.421). Additionally, these correlations were observed in the perihippocampal regions (left, r = −0.525; right, r = −0.517). In the posterior cingulate gyrus, the Z-score was positively and negatively correlated with blood flow in the left (r = 0.434) and right hemispheres (r = −0.445), respectively. These findings suggest that Z-scores obtained using the VSRAD are significantly correlated with the regional cerebral blood flow as measured by SPECT, which supports the clinical utility of the VSRAD in assessing the structural–functional relationships across multiple brain regions.

These results indicate that VSRAD-derived Z-scores are significantly associated with the perfusion changes detected by SPECT across multiple brain regions. This structural–functional correlation reinforces the clinical utility of the VSRAD as a complementary tool to cerebral blood flow SPECT.

#### 4.1.2. Comparison of Combined Use of the VSRAD and eZIS with Characteristic Findings of Dementia with Lewy Bodies

A study involving 42 patients with AD and 28 patients with DLB evaluated the utility of combining the VSRAD and cerebral blood flow SPECT [25]. This study used 99mTc-ECD as the radiotracer for SPECT imaging and eZIS for statistical analysis. Within eZIS, the Cingulate Island Sign (CIS) serves as a diagnostic biomarker for DLB. CIS refers to a pattern of preserved perfusion in the posterior cingulate gyrus despite marked hypoperfusion in the occipital lobes, which is an imaging feature that can differentiate DLB from other dementia types, especially AD.

The CIS score is calculated as follows:

(2) $$CISscore=\frac{Total\;Z\;score\;for\;decreased\;brain\;perfusion\;in\;the\;posterior\;cingulate\;gyrus}{Total\;Z\;score\;for\;decreased\;brain\;perfusion\;in\;the\;occipital\;lobe}$$

A CIS score ≥0.281 and <0.281 suggests a low and high likelihood of DLB, respectively [25]. Using the CIS score alone to differentiate between AD and DLB yielded a diagnostic accuracy of 84.6%, with a sensitivity of 92.3% and specificity of 76.9% [26]. The four diagnostic criteria for DLB were as follows: a CIS score on SPECT ≤ 0.259, a medial temporal lobe atrophy score on the VSRAD ≤ 2.05, a GM dorsal brainstem atrophy ratio ≥ 0.38, and a white matter dorsal brainstem atrophy ratio ≥ 0.42 [26]. The diagnostic accuracy for each criterion was as follows: 74% using the CIS score alone, 69% using only the medial temporal lobe atrophy score from the VSRAD, and 67% using only the dorsal brainstem atrophy ratios in the GM and white matter [26].

Taken together, these findings suggest that neither the VSRAD nor eZIS alone provides sufficient accuracy for distinguishing DLB from AD, but their combined use—incorporating both structural and perfusion markers—achieves higher diagnostic performance and supports clinical decision-making.

### 4.2. Characteristic Findings of MCI and AD and Their Comparison Using the VSRAD

A study involving 112 patients with MCI and 128 patients with AD evaluated the utility of the VSRAD and cerebral blood flow SPECT in differential diagnosis [27]. This study used 99mTc-ECD as the radiotracer for SPECT imaging. In eZIS, disease-specific ROIs were defined in the posterior cingulate gyrus/precuneus and bilateral parietal cortices. Three quantitative parameters were assessed.

1. Severity: defined as the sum of positive Z-scores on the hypoperfused side within the disease-specific ROI divided by the number of voxels showing positive Z-scores in the same region (normal ≤ 1.19);
2. Extent: calculated as the percentage of voxels with Z ≥ 2 on the hypoperfused side relative to the total number of voxels in the ROI (normal ≤ 14.2%);
3. Ratio: the extent of hypoperfusion in the disease-specific ROI divided by the extent of whole-brain hypoperfusion (normal ≤ 2.22) [28].

When comparing MCI and AD, the MMSE yielded an AUC of 0.835, which indicates a high discriminative accuracy. Contrastingly, the VSRAD yielded AUCs of 0.710 for the severity, 0.708 for the extent, 0.649 for the GM extent, and 0.677 for the ratio. The corresponding AUCs for eZIS were 0.616 (severity), 0.607 (extent), and 0.581 (ratio) [27]. Taken together, these findings suggest that using the VSRAD and eZIS as sole individual parameters has limited discriminative power for differentiating between MCI and AD. However, a multivariate logistic regression model that incorporated all these indices showed a significantly improved diagnostic performance. The following equation was used:

Pr(case) = 1/(1 + exp(−(13.1272 + 0.0244 × VSRAD VOI Extent + 0.0387 × eZIS Extent−0.5777 × MMSE)))(3)

This model had an improved AUC of 0.870 [27], which indicates that an enhanced diagnostic capability was achieved through the integrated analysis.

Another study involving 23 patients with DLB and 57 patients with AD evaluated the diagnostic utility of three imaging modalities: (A) the VSRAD, (B) eZIS using cerebral perfusion SPECT, and (C) 3D stereotactic surface projection using SPECT. Here, the Z-score of the left occipital lobe showed 80% accuracy as a diagnostic marker for distinguishing between DLB and AD [29].

The findings demonstrate that, although the VSRAD and eZIS parameters individually have limited discriminative power, combining them with cognitive measures such as the MMSE in a multivariate model markedly improves their diagnostic accuracy. Such integrated approaches illustrate the future potential of the VSRAD as part of a multimodal diagnostic workflow.

## 5. Comparison Between the VSRAD and Arterial Spin Labeling (ASL)

Okada et al. investigated the relationship between VSRAD scores and cerebral blood flow using ASL [30]. In 179 patients classified as being in the AD-type dementia group (mean age, 82.8 ± 5.7 years; males, 55; females, 125), weak negative correlations were observed between the percentage of total brain atrophy and the ASL maximum value at the posterior cingulate gyrus to the precuneus (r = −0.189 to −0.214; *p* < 0.01). Similarly, in 142 patients classified as being in the DLB-type dementia group (mean age 79.3 ± 7.79 years; males 65, females 15), a weak negative correlation was observed between the percentage of total brain atrophy and the ASL maximum value at the posterior cingulate gyrus to the precuneus (r = −0.125 to −0.233; *p* < 0.01).

These results demonstrated only weak correlations between the medial temporal atrophy measured by the VSRAD and the perfusion in the posterior cingulate gyrus and precuneus in both AD and DLB. The findings indicate a mismatch between structural atrophy and functional perfusion changes, highlighting the need for multimodal approaches and comparative studies between the VSRAD and other imaging modalities [30].

## 6. Combination VSRAD and Magnetic Resonance Spectroscopy (MRS)

A study evaluated the combined diagnostic utility of the VSRAD and MRS across multiple patient groups, including 93 control patients (mean age, 74.6 ± 10.2 years; males, 39; females, 54), 42 non-conversion patients with MCI (mean age, 78.9 ± 7.5 years; males, 19; females, 23), 25 conversion patients with MCI (after 3 years; mean age, 77.4 ± 7.1 years; males, 7; females, 18), 44 patients with AD (mean age, 80.6 ± 7.3 years; males, 18; females, 26), 8 patients with DLB (mean age, 74.0 ± 5.3 years; males, 3; females, 5), 5 patients with normal pressure hydrocephalus (mean age, 76.0 ± 4.2 years; males, 2; females, 3), and 11 patients with other neurological disease (mean age, 68.2 ± 12.5 years; males, 7; females, 4). Three key findings were identified as risk factors for AD after a 3-year follow-up: (1) increased medial temporal atrophy (MTA) severity on the VSRAD (MRI), (2) an increased choline/creatine ratio and myo-inositol/creatine ratio (1H-MRS), and (3) a decreased N-acetylaspartate/creatine ratio and N-acetylaspartate/myo-inositol ratio (1H0MRS) [31].

The VSRAD severity score at MTA demonstrated strong diagnostic performance, with an AUC of 0.90, sensitivity of 84.1%, and specificity of 84.6% at a cut-off value of 1.75 when distinguishing controls from patients with AD. For controls versus conversion MCI, the scores showed an AUC of 0.86, sensitivity of 85.7%, and specificity of 80.8%, at a cut-off value of 1.54 [31]. In contrast, the 1H0MRS ratio yielded an AUC of 0.75, sensitivity of 71.4%, and specificity of 71.2%, at a cut-off value of 1.77 for controls vs. AD, and an AUC of 0.77, sensitivity of 77.5%, and specificity of 70.2% at a cut-off value of 1.79 for control vs. conversion-MCI [31]. The VSRAD severity score at MTA/1H0MRS ratio showed an AUC of 0.94, sensitivity of 93.1%, and specificity of 82.2% at a cut-off value of 0.89 for controls vs. AD, and an AUC of 0.88, sensitivity of 85.7%, and specificity of 83.2% at a cut-off value of 0.90 for control vs. AD conversion-MCI [31]. These results suggest that combining the VSRAD and MRS improves the diagnostic accuracy for AD and conversion-MCI [31].

## 7. Applications of the VSRAD in Various Diseases and Research Contexts

To provide a comprehensive overview, the major clinical and research applications of the VSRAD reported in the literature are summarized in Table 1. This table outlines the key disease contexts, principal findings, and limitations, thereby facilitating a clearer understanding of the strengths and limitations of the VSRAD across diverse conditions.

### 7.1. Conversion from MCI to AD

A longitudinal study monitored 74 patients with MCI (29 males and 45 females; median age: 79.0 years). During the follow-up period, 29 patients (39.2%) progressed to AD, 39 (52.7%) remained at the MCI stage, and 6 (8.1%) regained normal cognitive function. Although there were no significant among-group differences in VSRAD scores, a trend was identified. Specifically, the Z-scores were highest in those who progressed to AD, intermediate in those who remained at the MCI stage, and lowest in those who regained normal cognitive function [32].

### 7.2. Comparison with Neuropsychological Assessments

A study including 18 patients with AD and 12 with MCI (mean age: 73.8 ± 8.26 years) reported a moderate positive correlation between VSRAD Z-scores and the Alzheimer’s Disease Assessment Scale–Cognitive Subscale (r = 0.488), which assesses cognitive dysfunction in patients with AD. Additionally, there was a significant negative correlation between Z-scores and the Wechsler Adult Intelligence Scale-III (r = −0.427) [33]. A community-based cohort study conducted in Seffuri Village, Saga Prefecture, Japan, examined the relationship between VSRAD scores and cognitive function in 213 cognitively healthy older adults (99 men and 114 women; mean age, 68.9 years). It observed negative correlations of hippocampal Z-scores with both the Rivermead Behavioral Memory Test (r = −0.37) and MMSE (r = −0.312) scores [34]. Another study involving 72 individuals (18 men and 54 women; 15 with AD, 12 with other dementia types, and 12 with MCI) investigated the association between Z-scores obtained using VSRAD Advance 2 and performance on the Cognistat cognitive assessment. Here, the Z-scores were negatively correlated with the “Orientation” (r = −0.35) and “Memory” subscale scores (r = −0.38) [35].

A study including 200 patients with clinical suspicion of cognitive impairment (48 males, 63 females; age: 46–94 years) observed a significant relationship of the right entorhinal cortex thickness with both the MMSE and HDS-R scores [36]. In 37 patients with DLB, the Z-score at medial temporal lobe atrophy showed a negative correlation MMSE and HDS-R scores [37].

### 7.3. Comparison with Executive Function Disorders and VSRAD

A study included 157 consecutive patients with AD or amnestic MCI and 107 patients with a CDR of 0.5 or 1. The participants were classified into three groups based on their hippocampal z-scores as follows: mild atrophy (0 < Z-score < 1.0, *n* = 21), moderate atrophy (1.0 ≤ Z-score < 2.0, *n* = 46), or severe atrophy (2.0 ≤ Z-score < 4.0, *n* = 40). There were significant among-group differences in the Frontal Assessment Battery (FAB) and its subtests, including similarities, lexical fluency, motor series, conflicting instructions, and go/no-go tasks. The mean FAB scores in each group were as follows: mild atrophy: 2.10 ± 0.94, moderate atrophy: 1.67 ± 0.67, severe atrophy: 1.29 ± 1.16 [38].

### 7.4. Evaluation of Brain Atrophy in Diabetes Mellitus

#### 7.4.1. Quantitative Assessment Using the VSRAD in Patients with Diabetes

A study utilized the VSRAD to assess hippocampal atrophy in 28 patients with type 2 diabetes mellitus (14 men and 14 women; mean age, 70.7 years) and 28 age- and sex-matched healthy controls [33]. The degree of hippocampal atrophy was quantified using the Hippocampal Region Atrophy Index (HAI), with a Z-score of ≥2 indicating significance. The mean HAI was significantly higher in the diabetic group (1.87 ± 1.08) than in the control group (1.22 ± 0.43). Furthermore, the Whole-Brain Atrophy Index, which represents the extent of whole-brain GM atrophy, was significantly higher in the diabetic group (8.63 ± 5.56) than in the control group (3.11 ± 1.95) [39].

#### 7.4.2. Relationship Between Visceral Fat and Hippocampal Atrophy in Patients with Diabetes

A study on 48 non-demented individuals with type 2 diabetes mellitus stratified the participants into two groups based on their visceral fat volume, i.e., the high- (*n* = 30; mean age, 65 years) and low- (*n* = 18; mean age, 65 years) visceral-fat groups. The mean Z-score in the hippocampal region was significantly higher in the high-visceral-fat group (1.366 ± 0.435) than in the low-visceral-fat group (0.539 ± 0.344), which suggests that increased visceral fat may be associated with greater hippocampal atrophy [40].

#### 7.4.3. Relationship Between Homocysteine Levels and Hippocampal Atrophy in Patients with Diabetes

A study involving 43 non-demented patients with type 2 diabetes mellitus who had MMSE scores ≥27 (26 men and 17 women; mean age: 65 ± 8 years) assessed hippocampal Z-scores using the VSRAD [41]. In this study, patients with elevated homocysteine levels had significantly higher mean Z-scores (1.336 ± 0.429) than those with low homocysteine levels (0.528 ± 0.338). This suggests a possible relationship between hyperhomocysteinemia and hippocampal atrophy in patients with diabetes.

#### 7.4.4. The Relationship Between Inflammation and Hippocampal Atrophy in Patients with Diabetes

A study on 45 patients with type 2 diabetes mellitus (26 men and 19 women; mean age, 65 ± 7 years) investigated the relationship between systemic inflammation and hippocampal atrophy [42]. VSRAD analysis revealed that patients with high-sensitivity C-reactive protein (hs-CRP) levels above the threshold had a significantly higher mean Z-score (1.221 ± 0.352) than those with hs-CRP levels below the threshold (0.479 ± 0.228). This suggests that systemic inflammation contributes to hippocampal atrophy in patients with diabetes.

#### 7.4.5. Relationship Between MMSE and VSRAD Scores in Elderly Patients with Diabetes

A study involving 67 elderly patients (≥65 years) with type 2 diabetes mellitus (43 men and 24 women; mean age, 73.0 years) assessed the relationship between cognitive function and hippocampal atrophy using the VSRAD [43]. A Z-score ≥2 in the hippocampal region was considered indicative of significant atrophy. In this study, the sensitivity and specificity for detecting cognitive decline using the MMSE alone were 80% and 48%, respectively. Contrastingly, the VSRAD alone showed sensitivity and specificity values of 100% and 89%, respectively. When the MMSE and VSRAD were combined, the sensitivity remained at 100%, while the specificity slightly increased to 90%.

### 7.5. Association Between Oral Health and VSRAD Scores

A study involving 15 participants (4 men and 11 women; mean age: 75.9 years) investigated the relationship between oral health and brain atrophy as assessed by the VSRAD [44]. First, their occlusal status was evaluated using the Eichner index, which classifies occlusal support based on tooth loss. The participants in Eichner group C had a significantly greater mean extent of GM atrophy (5.26 ± 1.22%) than those in Eichner groups A or B (3.41 ± 0.56%). When the participants were stratified based on their average number of remaining teeth (15.0 ± 11.1), those with fewer teeth than the mean had significantly greater atrophy (5.25 ± 1.22%) than those with more teeth (3.41 ± 0.56%). There was a significant negative correlation between the number of remaining teeth and GM atrophy (r = −0.549). Additionally, using the average masticatory function score (27.4 ± 8.49) as a threshold, those with a below-average score had significantly greater GM atrophy (5.09 ± 0.99%) than those with an above-average score (3.52 ± 1.02%). A significant negative correlation was found between masticatory function and GM atrophy (r = 0.432) [44]. Taken together, these findings suggest that a decreased tooth count and reduced masticatory function are associated with increased GM atrophy.

### 7.6. Association Between Olfactory Dysfunction and VSRAD Findings

A large-scale study involving 1383 participants (mean age: 75.1 ± 8.7 years) examined the association between cognitive function and olfactory ability [45]. The participants were classified into three groups: the dementia group (*n* = 567), intermediate cognitive group (*n* = 534; HDS-R score: 21–27, MMSE score: 24–27), and cognitively normal group (*n* = 283; HDS-R score ≥28, MMSE score ≥ 28). The participants’ olfactory function was assessed using the Japanese version of the University of Pennsylvania Smell Identification Test (UPSIT-J), which is scored on a 0–7 scale and involves four odorants (pepper, lemon, smoke, and strawberry). The correlation coefficients between the UPSIT-J scores and cognitive assessments were as follows: HDS-R, r = 0.567; MMSE, r = 0.532; and CDR, r = −0.5328, and all scores were statistically significant [45].

### 7.7. Association Between Driving Reaction and Medial Temporal Lobe Atrophy in Patients with MCI and AD

A driving simulation study included 11 individuals with MCI (mean age: 74.5 ± 6.4 years; seven men and four women) and 18 patients with AD (mean age: 75.8 ± 5.8 years; 11 men and eight women) [41]. The simulation utilized the Honda Safety Navi Version 2 driving simulator to evaluate three types of driving-related tasks:

1. Simple reaction task: Participants were instructed to press the accelerator pedal when a green light appeared. This task assessed reaction time and variability;
2. Choice reaction task: Participants were required to respond in accordance with the light color: brake and then accelerate for red, release and then press the accelerator for yellow, and continue pressing the accelerator for green. This task evaluated patients’ reaction time, its variability, and the number of errors that they made;
3. Divided-attention complex task: This task involved responding to both traffic lights and directional arrows displayed on the screen, which required the simultaneous use of both hands and the right foot. The instructions included pressing either the right or left button, depending on the direction of the arrow, or refraining from pressing if no arrow was shown;
4. In all tasks, CT was used to measure the operation time, variability, and error count. The AD group showed a significantly higher number of errors than the MCI group. The MMSE scores were negatively correlated with reaction time in the complex task (r = −0.3680) and error count in the divided-attention complex task (r = −0.4354) [46].

Subsequently, VSRAD analysis was conducted on 15 participants. The extent of GM atrophy was positively correlated with both reaction time (r = 0.5365) and its variability (r = 0.4617) in the simple task, as well as reaction time in the choice reaction task (r = 0.4887). Regarding specific VSRAD indices, the severity and extent of VOI atrophy were significantly correlated with the reaction time (r = 0.4807 and r = 0.4862, respectively) [46]. These findings suggested that medial temporal lobe atrophy, especially within the hippocampus, entorhinal cortex, and amygdala, may contribute to delayed reaction times while driving in individuals with cognitive impairment.

### 7.8. Investigation of Gut Microbiota, Cognitive Function, and VSRAD Findings

A study evaluated the effects of the probiotic Bifidobacterium on 115 participants with suspected MCI [47]. Initially, 130 participants were randomized into two groups: treatment (*n* = 65; 29 men and 36 women) and placebo (*n* = 65; 28 men and 37 women) groups. After 24 weeks, 55 (26 men and 29 women; mean age: 77.2 ± 5.8 years) and 60 (25 men and 35 women; mean age: 78.9 ± 4.3 years) individuals remained in the treatment and placebo groups, respectively. VSRAD analysis was performed on 42 participants (22 men and 20 women; mean age: 77.3 ± 6.1 years) and 47 participants (20 men and 27 women; mean age: 78.5 ± 4.2 years) in the treatment and placebo groups, respectively. In this double-blind, randomized controlled trial, evaluations were conducted at baseline as well as at weeks 8, 16, and 24. The extent of GM atrophy decreased (from 3.64 at baseline to 3.56 at 24 weeks) and increased (from 3.56 to 3.73) in the treatment and placebo groups, respectively. There was a significant between-group difference at 24 weeks [47], which suggests that probiotic intervention may suppress the progression of GM atrophy. Among the participants with baseline MMSE scores < 25, the MMSE subscore for temporal orientation substantially improved in the treatment group (from 4.5 to 4.87) but not in the placebo group (from 4.37 to 4.36). Taken together, these findings suggest that probiotic supplementation may improve certain aspects of cognitive function.

### 7.9. Depression and VSRAD Findings

In another study comparing 17 patients with late-onset depression (7 men and 10 women, mean age 77.5 years) and 21 patients with AD (8 men and 13 women, mean age 79.4 years), brain MRI evaluated by the VSRAD showed that volume loss in the right middle and inferior temporal gyri, uncus, posterior cingulate cortex, and precuneus was specific to patients with AD [48].

In a study on 71 patients with major depression (12 men and 59 women, age: 54–82 years), volume loss in the subgenual anterior cingulate cortex, evaluated using VSRAD Plus, was identified as a disease-specific change, with a sensitivity of 93%, specificity of 85%, and accuracy of 90% [49].

### 7.10. Semantic Dementia and VSRAD Findings

In a study on 29 patients with early-onset semantic dementia (age: 61.7 ± 4.5 years) and 39 patients with early-onset AD, a cut-off Z-score of 2.29 allowed differentiation between early-onset semantic dementia and AD, with a sensitivity of 87% and specificity of 85% [50].

### 7.11. Alcohol Consumption and VSRAD Findings

In a study involving 22 chronic alcoholic patients without neurological and psychological symptoms (mean age, 59.3 ± 4.1 years; MMSE, 28.5 ± 1.5; frontal assessment battery, 15.3 ± 1.2) and 22 control participants (mean age, 59.7 ± 3.9 years; MMSE, 29.0 ± 1.1; frontal assessment battery, 16.5 ± 0.6), VSRAD analysis revealed significant differences in parahippocampal gyrus atrophy. In the chronic alcoholic group, the Z-score at the parahippocampal gyrus was 1.88 ± 0.44, whereas the Z-score at the parahippocampal gyrus of the control group was 0.62 ± 0.29 [51].

### 7.12. Vitamin B12 Deficiency and VSRAD

Among 567 patients (mean age, 78.9 ± 8.5 years; 40% male, 60% female), 323 patients with a hippocampal atrophy Z-score of <2 (mean age, 78.3 ± 8.6 years; 126 males, 197 females) showed a significantly lower frequency of vitamin B12 deficiency compared with the other 244 patients, who had hippocampal atrophy Z-scores ≧ 2 (mean age, 79.9 ± 8.3 years; 102 males, 142 females) (4.6% vs. 12.2%, *p* < 0.01) [52].

### 7.13. Effectiveness of Non-Pharmacological Interventions Involving Physical Exercise in the Primary and Secondary Prevention of Dementia: VBM Reports

**Figure 2:**
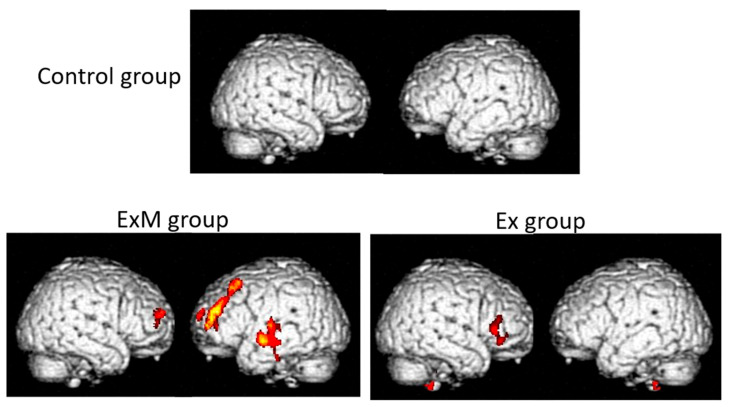
The results of the volumetric analyses using the VSRAD in the Mihama-Kiho scan project 1. The GM volumes before and after a year-long intervention were compared. The colored regions show the area where the GM volume showed a significant post-intervention increase.

Anatomical changes in the brain are among the biological markers for the primary and secondary prevention of dementia. It is important to be aware that the effectiveness of interventions for dementia should be determined using cognitive assessments. Nonetheless, neuroimaging data, including VBM results such as VSRAD parameters, show the intra-brain mechanism underlying improvements. However, non-pharmacological interventions cannot be considered as effective based only on neuroimaging findings, especially in the absence of significant improvements in cognitive assessments. The first report of the brain anatomical changes following interventions involving physical exercise was published in 2011 [53]. Image analysis was performed using the Oxford Centre for Functional MRI of the Brain (FMRIB)’s Integrated Registration and Segmentation Tool in FMRIB’s Software Library version 4.1, not the VBM. In this study, 120 participants (age: 50–80 years) were randomly divided into an aerobic exercise group and a stretching control group. Each group performed its respective physical activity led by a trained exercise leader. Neuropsychological and neuroimaging assessments were performed before and 1 year after the intervention. Specifically, volume changes in the hippocampus, thalamus, and caudate nucleus were analyzed. There were no between-group differences in the volumes of the thalamus and caudate nucleus. However, the aerobic exercise group showed an increase in hippocampal volume, which was related to improvements in spatial memory. Contrastingly, the volume of the stretching control group decreased similarly to that of the other two regions. The effectiveness of aerobic exercise for both primary and secondary prevention of dementia has been established [54]. It was suggested that the combination of aerobic exercise and cognitive stimulation (CS) therapy might be more effective than either intervention alone [55,56]. Since 2010, we have conducted a program for primary and secondary prevention of dementia using physical exercise combined with music (the Mihama-Kiho Project). In our first report on non-pharmacological interventions in community-dwelling older adults, we demonstrated that musical accompaniment enhanced the positive effects of physical exercise on cognitive function [57]. In that study, the first group (ExM) performed physical exercise (once a week for 1 h) with musical accompaniment, the second group (Ex) performed the same exercise without music, and the third group (Cont) received no intervention as the control. Before and after a year-long intervention, each participant was assessed using neuropsychological batteries. Physical exercise combined with music produced more positive effects on cognitive function than exercise alone, especially in visuospatial function (Mihama-Kiho Project Part 1). Neuroanatomical analysis using VBM revealed better preservation of the GM volume of the frontal lobe in the ExM group than in the Ex group, while it was decreased in the Cont group due to aging (Figure 2) (Mihama-Kiho Scan Project 1) [58]. These results indicate that, compared with exercise alone, physical exercise with music accompaniment induces greater effects on cognitive function and leads to neuroanatomical changes mainly at the frontal lobe in elderly people.

**Figure 3:**
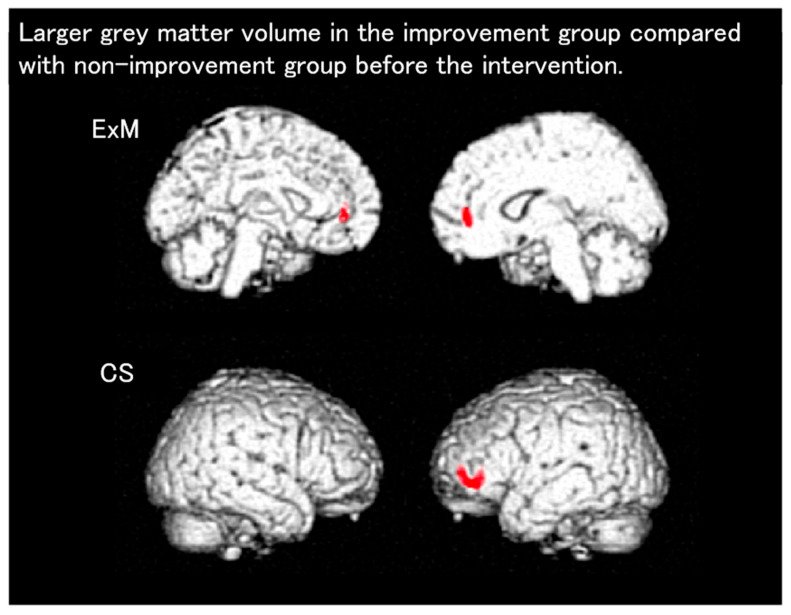
Results of the analyses using VBM in the Mihama-Kiho scan project 2. Colored regions show the area with significantly larger GM volume in the improvement group before the intervention. CS, cognitive stimulation; ExM, exercise with music accompaniment.

Next, we investigated the effects of physical exercise with music accompaniment on the cognitive function in patients with dementia (Mihama-Kiho Project Part 2) [59]. Patients with mild-to-moderate dementia participated in sessions of either physical exercise with music accompaniment or CS, once a week for 6 months. CS tasks such as drills, portable games, and calculations are often used in clinical and welfare situations for patients with dementia. Before and after the intervention period, neuropsychological and neuroimaging assessments were conducted. We performed between-group comparisons of the changes in cognitive function and GM volumes. Both groups showed significant post-intervention improvements in visuospatial function. Specifically, there were significant improvements in psychomotor speed and memory in the ExM and CS groups, respectively. The functional independence measure score, which reflects the activities of daily living, and the VSRAD scores were significantly preserved in the ExM group but significantly worsened in the CS group. Moreover, we investigated whether neuropsychological factors and regional brain atrophy measured via the VBM can predict the rate of post-intervention improvement in participants with mild-to-moderate dementia (Mihama-Kiho scan project 2) [60]. The participants were categorized into improvement (IMP) and no-IMP subgroups. VBM analysis revealed an increase in the GM volume of the anterior cingulate gyrus and left middle frontal gyrus among IMP participants in the ExM and CS groups, respectively, compared with no-IMP participants (Figure 3). These results suggest that some characteristics of pre-intervention regional brain atrophy may aid clinicians in determining the most appropriate non-pharmacological intervention for each participant.

The Mihama-Kiho Project Part 1 [57] had been planned to end within a year; however, the participants wanted to continue the ExM class. Therefore, they started to manage the class voluntarily and independently. Even now, 12 years after the end of the original study, the ExM class continues to take place with many active participants. In the Mihama-Kiho follow-up project, which was conducted 5 years after Part 1, we investigated the long-term effects of ExM by evaluating the cognitive function and GM volume in participants of the Mihama-Kiho Project Part 1 [57]. We compared the results of neuropsychological tests for participants who continued to take the ExM classes for 5 years with those of participants who did not. Additionally, we semi-quantitatively assessed the degree of atrophy in the parahippocampal gyrus using the VSRAD [61]. Analyses of the raw scores after the 5-year intervention revealed significant between-group differences in intellectual and memory function, with the ExM group performing better, as well as in the total and physical exercise subscores of the functional independence measure. Notably, the ExM group showed a significantly quicker psychomotor speed than the No-exercise groups. There was a significant correlation of the total number of ExM sessions attended with both the degree of changes in psychomotor speed (r = −0.22, *p* = 0.02) and scores of memory function (r = −0.20, *p* = 0.036) (VSRAD was insignificant: r = −0.054, *p* = 0.59). This is the second study to assess the long-term effects of a 5-year intervention of physical exercise. Moreover, this is the first study to attempt to assess long-term changes in the volume of the parahippocampal gyrus using MRI. Morphometric analysis is usually used to diagnose diseases such as AD and assess post-intervention anatomical changes. VBM and the VSRAD may be powerful tools for elucidating brain changes.

### 7.14. VSRAD Findings in Patients with HIV (Human Immunodeficiency Virus)

A study on 40 relatively young patients with HIV and 30 age-matched controls revealed significant between-group differences in whole-brain GM atrophy, with the patients with HIV showing greater GM atrophy [62].

### 7.15. VSRAD Findings in Patients Undergoing Hemodialysis

A study on 34 patients undergoing hemodialysis (17 men and 17 women, age: 68.4 ± 7.34 years) revealed a correlation of VSRAD-measured hippocampal atrophy with total serum homocysteine levels and age [63].

### 7.16. Finger Function and VSRAD Finding

A study on 62 patients with AD (24 men and 38 women, age: 76.7 ± 7.7 years) revealed a significant correlation between the Z-score for medial temporal lobe atrophy and the Montreal Cognitive Assessment Japan score (r = −0. 52; *p* < 0.001) [64]. After the a finger tapping-movement test, the standard deviation of the distance rate of velocity peak in extending movements in the non-dominant hand showed a significant correlation with the severity of medial temporal lobe atrophy (r = 0. 51; *p* < 0.001) [64].

### 7.17. Eye Movement and VSRAD Findings

A study on 300 individuals (84 with AD (age: 75.6 ± 16.3 years), 126 with MCI (age 74.1 ± 16.1 years), and 90 who were healthy 73.8 ± 16.4 years) reported significant correlations of responsive search scores from exploratory eye movement with age, HDS-R, MMSE, and VSRAD scores (age; r = −0.159/−0.147/−0.173, HDS-R; r = 0.512/0.249/0.256, MMSE; r = 0.477/0.234/0.386, VSRAD; r = −0.428/−0.274/−0/391) [65].

### 7.18. Alcohol and VSRAD Finding

In a study on 22 chronic alcoholic patients (age, 59.3 ± 4.1; MMSE, 28.5 ± 1.5; frontal assessment battery, 15.3 ± 1.2) without neurological and psychological symptoms and 22 control patients (age, 59.7 ± 3.9; MMSE, 29.0 ± 1.1; frontal assessment battery, 16.5 ± 0.6), in the chronic alcoholic group, the Z-score at the parahippocampal gyrus was 1.88 ± 0.44 and, in the control group, the Z-score at the parahippocampal gyrus was 0.62 ± 0.29 [51]. From these results, it can be determined that there is atrophy at the parahippocampal gyrus in the chronic alcoholic group.

## 8. VSRAD and Artificial Intelligence

### Artificial Intelligence and VSRAD

In a study involving 7932 patients (mean age, 77.43  ±  9.71 years; males, 2977; females, 4955) diagnosed with AD, other dementias, MCI, or psychiatric disorders or classified as cognitively normal, the utility of combining an artificial intelligence (AI)-based AD diagnostic model and the VSRAD was reported [66]. The AI-based AD diagnostic model was developed using 6000 training data and 1932 test data. The model showed an AUC of 0.819 (95% CI, 0.797–0.840) when combined with the VSRAD, and an AUC of 0.817 (95% CI, 0.795–0.8390) without the VSRAD for AD diagnosis. The VSRAD alone showed an AUC of 0.717 (95% CI, 0.691–0.742) [66].

## 9. Limitations

This study has several limitations. First, the use of the VSRAD is restricted since it exclusively relies on data from Japanese individuals, which limits its utility in other countries. Secondly, there have been no large-scale, multicenter studies to support the broader validity and reliability of the VSRAD. Third, there are limited comparative studies between medical imaging and pathological confirmation, which limits its diagnostic accuracy, especially in distinguishing between AD and DLB. Although the VSRAD may facilitate this differentiation, the Hisayama Study—a large cohort study conducted in Japan—revealed that mixed cases of AD and DLB are common, with this combination tending to worsen the severity of dementia [67]. We believe that the VSRAD has insufficient sensitivity and specificity for detecting DLB, and thus should be used with caution. Fourth, the utility of the VSRAD for distinguishing between AD and hippocampal atrophy in frontotemporal lobar degeneration is limited [68]. Fifth, the comparative evaluations between the VSRAD and other imaging modalities are insufficient. Further research is necessary to compare the relative performance of the VSRAD with that of single-photon emission tomography or positron emission tomography.

Finally, the role of the VSRAD in clinical practice remains to be established. Recent reports have highlighted the utility of amyloid positron emission tomography in managing patients with MCI [69]. It remains necessary to determine whether the VSRAD should be applied for screening, assessing treatment efficacy, or monitoring follow-up. Additionally, artificial intelligence should be incorporated in the development of the VSRAD.

## 10. Conclusions

Quantifying brain atrophy using the VSRAD allows objective evaluation and facilitates the investigation of its association with various diseases. Specifically, the VSRAD can be considered a useful adjunctive tool for diagnosing AD and DLB.

## Figures and Tables

**Table 1:** Clinical and research applications of the VSRAD. Summary of representative diseases and conditions in which VSRAD has been applied, along with key findings and limitations reported in the literature.

Disease/Condition	Key VSRAD Findings	Limitations
mer’s disease (AD)	High diagnostic accuracy (>90%); hippocampal/parahippocampal atrophy correlates with severity	Underestimates early-onset AD; limited generalizability outside Japan
Mild cognitive impairment (MCI)	Higher Z-scores in converters to AD; correlation with MMSE/memory tests	Predictive value moderate; overlap with aging
Dementia with Lewy bodies (DLB)	Lower entorhinal cortex Z-scores vs. AD; dorsal brainstem indices useful	Sensitivity/specificity moderate; overlap with AD
Diabetes mellitus	Hippocampal and whole-brain GM atrophy; linked with visceral fat, homocysteine, hs-CRP	Cross-sectional; causality unclear
Oral health	Fewer teeth/reduced masticatory function → more GM atrophy	Small sample; cross-sectional
Olfactory dysfunction	Hippocampal Z-scores correlated with impaired smell identification	Confounding factors not fully addressed
Depression	Specific atrophy in subgenual ACC; distinct from AD	Small samples; partial overlap with AD
Semantic dementia	Z-score cut-off differentiates from AD (sens. 87%, spec. 85%)	Needs larger validation
Alcoholism	Parahippocampal atrophy in chronic alcoholics	Confounding by nutrition, comorbidities
Vitamin B12 deficiency	Higher prevalence of deficiency in patients with Z ≥ 2	Observational only
HIV infection	Greater GM atrophy in younger HIV patients	Not disease-specific
Hemodialysis	Hippocampal atrophy correlates with homocysteine/age	Pilot study
Interventions (exercise, probiotics, AI)	Exercise+music preserved GM; probiotics slowed atrophy; AI models improved AUC	Heterogeneous methods; limited long-term data
VSRAD =The Voxel-Based Specific Regional Analysis System for Alzheimer’s disease; MCI = mild cognitive impairment;		
DLB = dementia with Lewy bodies; AD = Alzheimer’s disease; PSP = progressive supranuclear palsy; DB = database.		
AD = Alzheimer’s disease; MCI = Mild cognitive impairment; DLB = Dementia with Lewy bodies; DM: Diabetes mellitus;		
GM = Gray matter; MMSE = Mini-Mental State Examination; ACC = Anterior cingulate cortex; ACC = Anterior cingulate cortex;		
MDD = Major depressive disorder; LOD = Late-onset depression; SD = Semantic dementia; HIV = Human immunodeficiency virus;		
HD = Hemodialysis; AI = Artificial intelligence; AUC: Area under the curve.		

## Data Availability

No new data were created or analyzed in this study. Data sharing is not applicable to this article.

## Abbreviations

The following abbreviations are used in this manuscript:

AD	Alzheimer’s disease
CDR	Clinical Dementia Rating
CT	Computed tomography
DARTEL	Diffeomorphic anatomical registration through exponentiated lie algebra
DICOM	Digital Imaging and Communications in Medicine
DLB	Dementia with Lewy bodies
DTI	Diffusion tensor imaging
DWI	Diffusion-weighted imaging
fMRI	Functional MRI
GM	Gray matter
HDS-R	Hasegawa Dementia Scale
MMSE	Mini-Mental State Examination
MRI	Magnetic resonance imaging
ROI	Regions of interest
SPECT	Single-photon emission computed tomography
SPM	Statistical parametric mapping
VBM	Voxel-based morphometry
VOI	Volume of interest
VSRAD	Voxel-based specific regional analysis system for Alzheimer’s disease
